# Targeting the inhibitory receptor CTLA-4 on T cells increased abscopal effects in murine mesothelioma model

**DOI:** 10.18632/oncotarget.3487

**Published:** 2015-03-08

**Authors:** Licun Wu, Matthew Onn Wu, Luis De la Maza, Zhihong Yun, Julie Yu, Yidan Zhao, John Cho, Marc de Perrot

**Affiliations:** ^1^ Latner Thoracic Surgery Research Laboratories and Division of Thoracic Surgery, Toronto General Hospital, University Health Network, University of Toronto, Toronto, ON, Canada; ^2^ Radiation Oncology, Princess Margaret Hospital, University Health Network, University of Toronto, Toronto, ON, Canada

**Keywords:** mesothelioma, local radiotherapy, abscopal effect, immune checkpoint

## Abstract

We previously demonstrated that blockade of immune suppressive CTLA-4 resulted in tumor growth delay when combined with chemotherapy in murine mesothelioma. Tumor-infiltrating T cells (TIT) after local radiotherapy (LRT) play critical roles in abscopal effect against cancer. We attempt to improve the local and abscopal effect by modulating T cell immunity with systemic blockade of CTLA-4 signal. The growth of primary tumors was significantly inhibited by LRT while CTLA-4 antibody enhanced the antitumor effect. Growth delay of the second tumors was achieved when the primary tumor was radiated. LRT resulted in more T cell infiltration into both tumors, including Treg and cytotoxic T cells. Interestingly, the proportion of Treg over effector T cells in both tumors was reversed after CTLA-4 blockade, while CD8 T cells were further activated. The expression of the immune-related genes was upregulated and cytokine production was significantly increased. LRT resulted in an increase of TIT, while CTLA-4 blockade led to significant reduction of Tregs and increase of cytotoxic T cells in both tumors. The abscopal effect is enhanced by targeting the immune checkpoints through modulation of T cell immune response in murine mesothelioma.

## INTRODUCTION

Malignant pleural mesothelioma (MPM) usually spreads locally along the ipsilateral pleura and distant metastasis is typically seen at the advanced stages [1]. Nowadays, radical surgery and hemithoracic high dose radiation have shown encouraging results and is employed as primary forms of treatment in some cancers [2-4]. Surgical options include debulking of the pleura by pleurectomy/decortication (P/D) or a more aggressive extrapleural pneumonectomy (EPP) involving removal of the lung, diaphragm, and involved pericardium [2,3]. Even after receiving the radical surgery, MPM almost always recurs and/or metastasizes to the counterlateral chest or abdomen, resulting in poor prognosis [3,4]. Therefore, combinations of chemotherapy, surgery, and radiation therapy were initiated as a new treatment strategy to improve prognosis [5]. Advances in radiation technique have allowed the administration of high dose hemithoracic radiation therapy before or after surgery [6]. The ideal timing of chemotherapy relative to surgery and the role of intracavitary chemotherapy continue to be controversial issues [7].

Radiotherapy can induce direct cancer cell death while also producing a systemic anti-tumor effect in the non-irradiated field, which has been referred to as the abscopal effect [8]. Abscopal effect is a systemic effect mediated by activation of anti-tumor immune responses by local radiotherapy, which can convert the irradiated tumor into an in situ vaccine. Evidence has shown that the radiotherapy triggers cytotoxic T cell amplification and dendritic cell activation in several types of cancer [9]. One explanation why local radiation may help stimulate the immune system to combat cancer is based on stimulation of T cells by the induction of lethal DNA damage in tumor cells and the induction of immunogenic cell death [10]. A short course of high dose local radiotherapy (LRT) administered to established tumors can lead to increased T cell priming and T cell dependent tumor regression. It has been shown that type I interferon (such as IFN-γ) plays an essential role in LRT-mediated tumor control. LRT drastically enhances the cross-priming capacity of tumor-infiltrating dendritic cells (DC) from wild-type mice but not IFN-γ receptor-deficient mice or immunodeficient mice [11]. The enhanced cross-priming ability of DCs after LRT was dependent on autocrine production of IFN-γ. A short course of high-dose of LRT can trigger production of IFN-γ that initiates a cascading innate and adaptive immune targeting of the tumor [11]. Another study showed that chemoradiotherapy may induce immunogenic cell death, which could trigger T cell immunity mediated by high mobility group box-1 protein (HMGB-1) and calreticulin [12].

The abscopal effect has been emphasized recently based on some evidence that metastasis regressed after local radiotherapy in several cancer types [13-15]. However, the biological mechanisms underlying the phenomenon are not fully understood, but more and more evidence supports a notion that it is mediated by immunologic mechanisms [16-18]. Our previous study and others have demonstrated that systemic blockade of the immunosuppressive CTLA-4 is able to enhance the immune responses against tumor [19-21]. Cyototoxic T lymphocyte-associated antigen-4 (CTLA-4), also known as CD152, is a member of the CD28/B7 immunoglobulin superfamily of immune regulatory molecules. CTLA-4 shares its 2 ligands (B7-1 and B7-2) with its costimulatory counterpart CD28. CTLA-4 and CD28 and their ligands B7-1 (CD80) and B7-2 (CD86) are critically important for the initial activation of naive T cells and regulation of the clonal composition of the responding repertoire following migration of activated dendritic cells to lymphoid organs. In murine breast cancer model, anti-CTLA-4 mAb alone failed to induce effective immune response to poorly immunogenic tumors but successful when combined with local ionizing radiation therapy [21]. Wirsdorfer et al found that thorax irradiation resulted in local and systemic accumulation of Treg cells expressing surface proteins characteristic for recruitment and immunosuppressive activity (CD103, CTLA-4 and CD73) [22]. A recent study showed that X-ray irrdiation combined with anti-CTLA4 monoclonal antibody can improve the antitumor effect and prolong median survival time in murine lymphoma and Lewis lung cacner [23]. We studied the abscopal effect induced by LRT with γ-ray irradiation in murine mesothelioma model and determine if this effect could be promoted by removing the immunosuppressive CTLA-4 signal.

## RESULTS

### Surviving fraction of cultured AB12 cells after exposure to γ-ray radiation

Cultured AB12 cells were exposed to serial doses of γ-ray radiation from 0 to 20Gy. Clonogenic assay showed that the surviving fraction at a dose of 5Gy is approximately 10%, whereas the cells exposed to 10Gy or higher doses lost the ability to form colonies (Supplementary Fig. 1A & 1B). Radiation-induced cellular apoptosis and death were evaluated by Annexin V and Flour-450, respectively, and the effect was dose dependent (Supplementary Fig. 1C & 1D). Therefore, 5Gy was selected as optimal dose in animal model.

### LRT resulted in growth delay of both primary and secondary tumors, and addition of CTLA-4 blockade enhanced the antitumor effect

**Figure 1:**
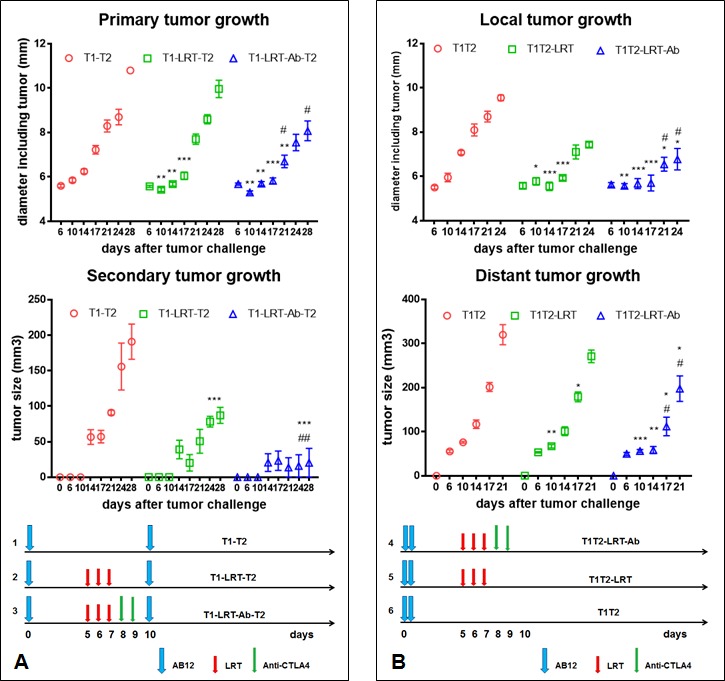
The effect of T1 local radiation on the growth of T2 tumors in the absence or presence of systemic CTLA-4 blockade A) The growth of primary and secondary tumors in sequential models. At day 10 after tumor cell injection of the primary site, the secondary tumor was challenged. LRT 5Gy was initiated on day 5 after tumor challenge onto the right thigh, and CTLA-4 blockade with its monoclonal antibody was given after completion of radiation (left diagram). The growth of primary (T1) and secondary tumor (T2) was shown in the middle and right panel, respectively. B) The growth of local and distant tumors in concurrent models. Two tumors T1 and T2 were challenged concurrently on day 0. Treatment schedule was shown in the diagram (bottom panel) and effect on tumor growth was shown in the top and middle panels. * represents comparison with untreated group and # represents comparison with LRT alone group, respectively.

The growth of primary tumors slowed down after local radiation, and the addition of CTLA-4 blockade improved the effect induced by radiation (Fig. 1A & 1B, middle panels). Interestingly, the secondary or distant tumors of mice whose primary tumor was treated with LRT grew slower than those of untreated mice. More strikingly, the Anti-CTLA4 following LRT gave rise to significant growth delay, and in the sequential model, some of the secondary tumors (3/9) were completely rejected (Fig. 1A & 1B, right panels), while treatment with Anti-CTLA4 alone had no effect on tumor growth.

For the immune deficient NOD/SCID mice, however, local radiation had some effect on the primary tumor growth, but less effective than in wild type Balb/c mice. The growth of distant tumors was not significant different between LRT-treated and untreated groups (Supplementary Fig. 2). In another control experiment, only one tumor was injected into the right flank (T2) of Balb/c mice, the delivery of LRT to the right leg (T1 free) did not result in any growth delay of the tumor on the right flanks (Supplementary Fig. 2).

### Effect of irradiated AB12 cell vaccine on the distant tumor growth and on T cell response

**Figure 2:**
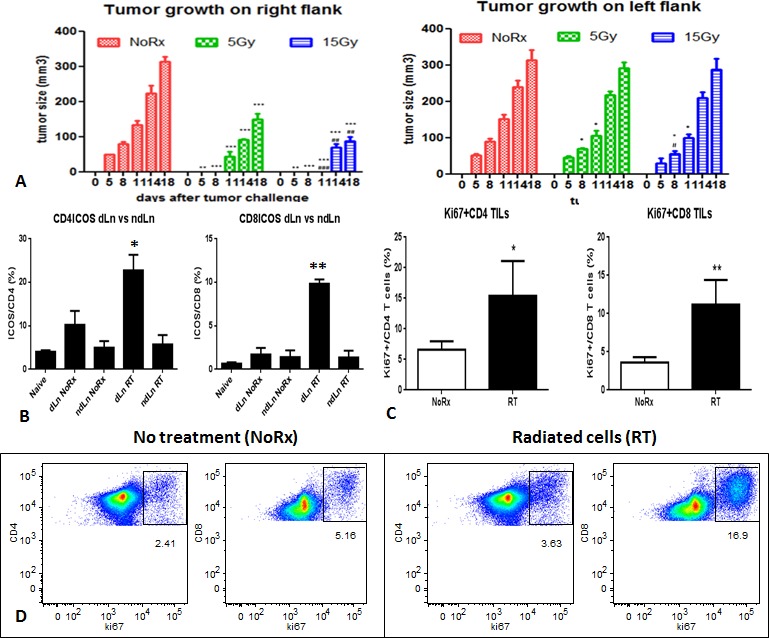
A) Primary tumor development after injection of irradiated AB12 cells, and the effect on the growth of distant tumors on the left flanks; B) Activation of CD4 and CD8 T cells in the draining lymph nodes derived from mice that were injected with the irradiated AB12 cells versus untreated cells; C) Proliferation of tumor-infiltrating T cells was dramatically increased in the radiated group, especially CD8 T cells; D) T cell proliferation of splenocytes after overnight culture was also increased after irradiated AB12 cell vaccination.

The growth of tumors on the right flank of mice injected with 5Gy or 15Gy irradiated cells was significantly slower than that of the non-radiated group. On the counterlateral site, injection of non-radiated cells led to tumors that grew significantly more slowly in mice receiving cells radiated with 5Gy or 15Gy on the right side. However, the effect appeared to be transient and unable to inhibit tumor growth beyond 11 days (Fig. 2A).

On day 10 after tumor challenge mice were sacrificed to assess T cell response in the tumor, draining lymph node and spleen. It was found that the expression of ICOS in both CD4 and CD8 T cells increased significantly in the draining lymph nodes 10 days after injection of the radiated cells, compared with the non-radiated cell injection (Fig. 2B). The proliferation of tumor-infiltrating T cells, especially CD8 T cells increased significantly 10 days after injection of the radiated cells (Fig. 2C). Similar phenomenon was found in the draining lymph nodes, however, no significant difference was observed in the spleen (Supplementary Fig. 3). The proportion of dendritic cells in the draining lymph nodes was found to increase (Supplementary Fig. 4). After overnight culture of the splenocytes, the proliferation of total T cells, CD4 and CD8 T cells was observed to increase in the mice with radiated cell injection, and the percentage of total T cells tripled, and a dramatic increase in CD8 T cells was notable (Fig. 2D).

### Enhancement of T cell infiltration into the tumor after local radiation and CTLA-4 blockade

**Figure 3:**
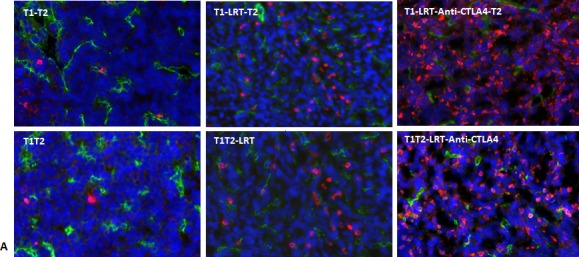

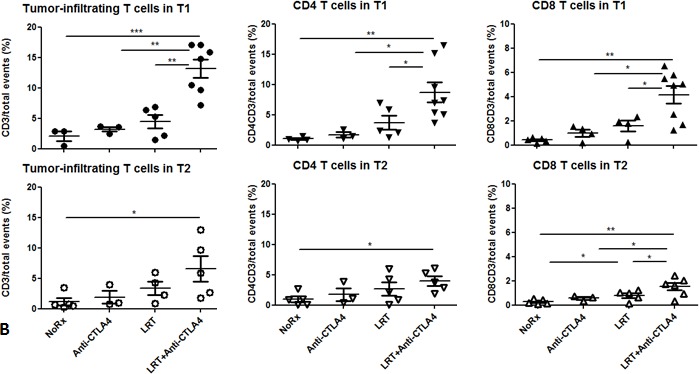
T cells infiltrated into tumors T1 and T2 in both models A) Total T cells infiltrated into tumors were recognized by CD3-Alexa555 conjugated antibody (red), blood vascular endothelial cells were stained by CD31-Alexa488 (green), and all nuclei were stained by DAPI (blue), 400×. Representative images were acquired from T1 of each group in both models; B) Tumor-infiltrating T cells including total T cells and CD4^+^ and CD8^+^ T cells were quantified by flow cytometry. Tumor tissues were collected at 7 days after completion of treatment. Data shown were generated from T1 and T2 in the sequential model. The experiment was repeated twice.

Fluorescent immunostaining demonstrated that more T cells infiltrated into the tumor T1 after local radiation combined with CTLA-4 blockade, whereas only a few T cells were observed to infiltrate into the untreated tumors (Fig. 3A).

Quantification of tumor-infiltrating T cells by flow cytometry showed that the percentages of total T cells, and CD4^+^ and CD8^+^ T cells were higher in LRT followed by CTLA-4 blockade in both T1 and T2 (Fig. 3B).

### Increase of activated CD4 and CD8 T cells in spleen and decrease of Treg cells infiltrated into tumor after local radiation followed by systemic blockade of CTLA-4

**Figure 4:**
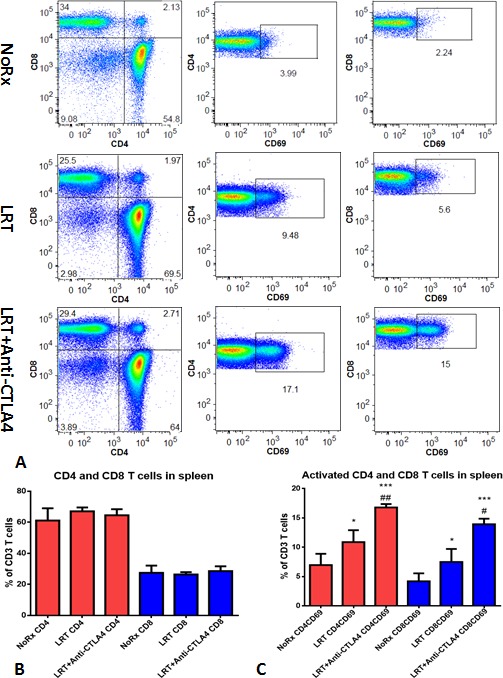

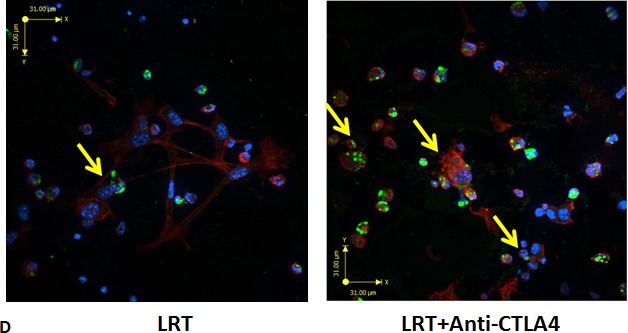
Total and activated CD4 and CD8 T cells from the splenocytes after overnight culture A representative result from treatment with LRT versus LRT+Anti-CTLA4 compared with the untreated group was shown (A); Total CD4 and CD8 T cells had no significant difference (B), whereas the activated CD4 and CD8 T cells increased significantly in the group treated with LRT+Anti-CTLA4 compared with LRT alone (C). * represents comparison with untreated group and # represents comparison with LRT alone group, respectively. Comparison of *in vitro* cell killing of splenocytes derived from mice treated with LRT alone and LRT in combination with anti-CTLA4 mAb (D). A representative image shows the co-culture of splenocytes and target cells at a ratio of effector:target=20:1, resulting in tumor cell lysis after overnight culture in 2ml RPMI1640 complete medium in a 24-well plate. Blue: DAPI, Red: Actin, and Green: CD8 T cells.

On day 20 after completion of radiation in the sequential setting (T1-T2), the splenocytes were cultured 5h to evaluate T cell activation. The percentages of total T cells and CD4/CD8 T cells in the spleen did not change dramatically in the group LRT versus LRT combined with CTLA-4 blockade, when compared with untreated group. However, the percentage of activated CD4 and CD8 T cells increased significantly in the combination group, especially activated CD8 T cells were almost three-fold higher than after LRT alone (Fig. 4A-C).

Co-culture of tumor cells and splenocytes derived from mice treated with LRT combined with anti-CTLA4 mAb displayed more cytotoxic T cells and more frequent cytolytic target cells, compared with those from the LRT alone group (Fig. 4D).

**Figure 5:**
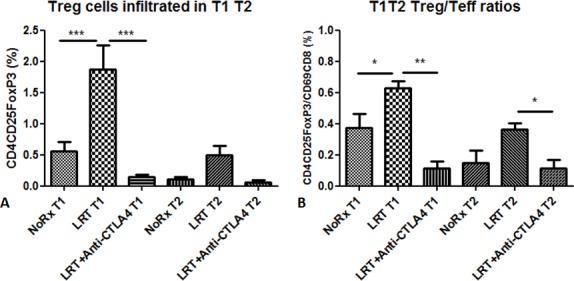
Treg cells infiltrated into the tumors (T1 and T2) 7 days after completion of local radiation in the absence or presence of administration with anti-CTLA4 antibody Proportion of tumor-infiltrating Treg cells was presented as percentage in total acquired events (A); Ratios of Treg cells to activated T cells in T1 and T2 (B).

The percentage of CD4^+^CD25^+^FoxP3^+^ Treg cells and the ratio of Tregs to effector CD8 T cells were found to increase in both tumors on day 7 after treatment with LRT, and this phenomenon was reversed by treatment with CTLA-4 blockade (Fig. 5).

### The expression of the immune-associated genes and cytokine production after treatment with LRT and CTLA-4 blockade

**Figure 6:**
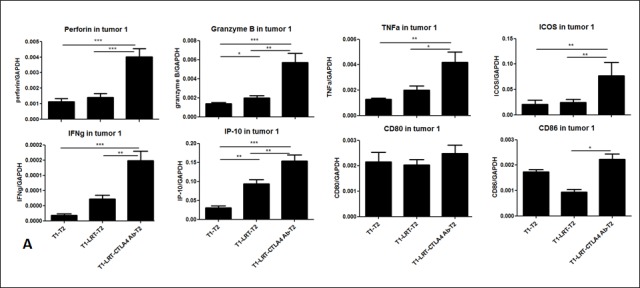

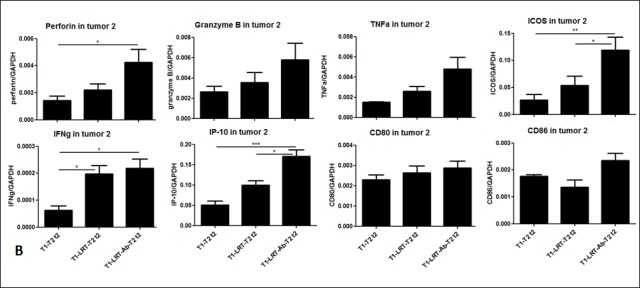

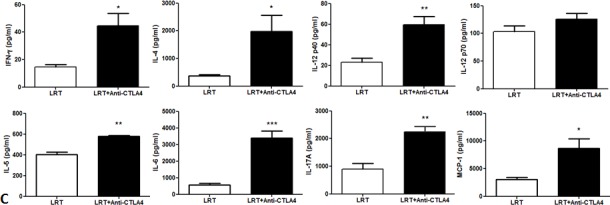
The expression of the immune-related genes was evaluated by RT-PCR in tumor T1 (A) and T2 (B); and the production of cytokine profile was determined by Luminex assay, where the concentrations are shown in pg/ml of culture medium (C).

RT-PCR results demonstrated that LRT combined with anti-CTLA-4 antibody resulted in upregulation of the immune-associated genes such as IFN-γ and its inducible protein perforin IP-10, cytolytic enzymes perforin and granzyme B, inducible costimulation molecule ICOS, DC maturation markers CD80 and CD86. This occurred in both T1 and T2 tumors compared with LRT alone or untreated tumors (Fig. 6A & 6B).

Cytokine profile determined by Luminex assay showed that the levels of IFN-γ, IL-4, IL-5, IL-6, IL-12p40 and p70, IL-17A, and MCP-1 in the supernatant of cultured splenocytes was higher in the group treated with LRT followed by CTLA-4 blockade than those of LRT alone (Fig. 6C).

## DISCUSSION

In order to perform local radiotherapy appropriately in a mouse model, the radiation source must be focused on the tumor precisely while the rest of the body is protected from scattered radiation. Tumor cells were injected into the right hind leg so as to make it feasible for local radiation. A lead box was initially made of 5-layer lead shield (each layer 1/32 inch), and the tumor-bearing leg was exposed to the radiation. However, severe systemic toxicity was observed as measured by the rapid decrease in the total number of T cells. An especially dramatic reduction of CD8 T cells was observed and led to the rise of CD4/CD8 T cell ratio [24]. Animals were visibly sick and passed away within two weeks (unpublished data). Following this, we constructed a lead chamber capable of protecting the body sufficiently from the scattered radiation. Mice receiving local radiation were active during the experimentation. Total T cells and the CD4/CD8 T cell ratio are not statistically different from naïve mice. All the following experiments were performed using this lead chamber.

As demonstrated previously, local radiation can induce tumor cell death directly, and dead tumor cells release a wide variety of tumor-associated antigens which are captured by dendritic cells to give rise to cross presentation [25]. To some extent, LRT-induced tumor cell death and tumor vaccination play similar roles in inducing immunogenic tumor cell death within a tumor microenvironment by stimulating immune cells [9,26]. We injected irradiated AB12 cells with 5Gy or 15Gy into the right flanks of mice and non-irradiated cells into the counterlateral flank, and studied the induction of systemic immune activation. We observed that the irradiated tumor cell vaccine induced systemic immune effects resulting in growth delay of distant tumors (Fig. 2). We then injected tumor cells to the right hindleg and radiated them *in vivo* with 15Gy in 3 fractions and observed the effect in the radiated and distant tumros. The effect of non-irradiated tumors, known as the abscopal effect, can be attributed to the activation of an antitumor immune response [8]. This effect was observed directly in our model.

Besides local activities, the abscopal effect's impact on non-irradiated tumor growth originates at least in part from enhanced immune functions such as DC and cytotoxic T lymphocyte (CTL) activation. DCs present the tumor peptides via MHC complexes and deliver co-stimulatory signals to naïve CD4 and CD8 T cells. The latter differentiate into CTLs that mediate tumor-specific cell killing [27]. Therefore, combination of radiation with further immune modulation may enhance the abscopal anti-tumor immune reaction.

In this study, we designed two experimental settings either sequential or concurrent challenge of two tumors. For the sequential model, CTLA4-based immunotherapy was given 1 day after completion of radiation, and the second challenge was delivered. The idea of LRT followed by immunotherapy was developed after completion of an innovative clinical study for MPM patients with a short accelerated course of high-dose hemithoracic intensity-modulated radiation therapy followed by EPP. This phase I/II study assessed the feasibility of Surgery for Mesothelioma After Radiation Therapy (SMART) and showed that the cumulative 3-year survival reached 84% in epithelial subtypes compared with 13% in biphasic subtypes [28].

*In vitro* data demonstrated that a single dose of radiation led to tumor cell apoptosis and death. LRT-induced tumor cell death as in situ vaccination produced a systemic immune response against tumors in the primary and secondary sites. Consecutive 3 doses of radiation resulted in tumor growth delay and the effect was enhanced significantly by addition of CTLA-4 blockade (Fig. 2). This finding might be able to explain why pre-operative radiotherapy has greater clinical outcomes, indicating that the abscopal effect could prevent potential tumor recurrence or metastasis. On the other hand, for the concurrent setting, LRT-induced systemic effects could delay the growth of distant tumors and this effect could be promoted by the CTLA-4 blockade.

The lack of specific tumor antigens in mesothelioma makes it difficult to monitor specific immune response, interefering with the development of novel targeted therapies [29]. However, the irradiated tumor cells killed by LRT can be converted to an in situ vaccine, which includes all tumor-associated antigens and thus is tumor-specific [30]. Cytotoxic and cytolytic cytokines were detected to evaluate the overall immune response. Cytokine cascade as a response to LRT may be one of the mechanisms to account for the abscopal effect. The release of cytokines is able to augment tumor immune surveillance and inhibit tumor growth through direct tumoricidal properties [31]. Robust clinical associations between cytokine release and abscopal regression of tumor in the scenario of local radiation are yet to be established. However, in the preclinical setting, cytokine activation has been demonstrated in association with distant organ effects. When rat lung was partially irradiated, micronucleus formation was observed in other non-irradiated areas of the lung, indicating DNA damage at these non-irradiated sites [32]. This was accompanied by macrophage activation and production of cytokines, lasting up to 16 weeks after irradiation [33].

In the recent years, the immune checkpoints in tumor microenvironment have become new treatment paradigms. Strategies to block these checkpoints have shown promising perspectives in cancer immunotherapy [20,34,35]. However, there are only few studies concerning checkpoint blockade in the context of mesothelioma immunotherapy [19,20]. We are optimistic that the blockade of immune checkpoints combined with conventional therapy especially following local radiotherapy will be able to improve the effectiveness of mesothelioma treatment.

Taken together, the growth delay of both primary tumors (T1) and secondary tumors (T2) was achieved in mice whose primary tumor was treated with LRT, while the addition of CTLA-4 antibody enhanced the radiation-induced antitumor effect. LRT resulted in more T cell infiltration into both primary and secondary tumors. Further, the expression of the immune-related genes was upregulated and cytokine production was significantly increased after treatment with LRT+Anti-CTLA4 compared with LRT alone. Interestingly, LRT resulted in an increase of tumor-infiltrating Tregs, while CTLA-4 blockade gave rise to reversal of Tregs in both tumors. LRT of primary tumors displayed abscopal effect on the secondary or distant tumor, and this effect was enhanced by systemic blockade of the immune checkpoint through modulation of T cell immune response in murine mesothelioma.

## MATERIALS AND METHODS

### Murine mesothelioma cell line and tumor implantation into mice

Murine mesothelioma AB12 cells were cultured in RPMI 1640 medium supplemented with 10% fetal bovine serum and 1% penicillin and streptomycin, and maintained at 37°C in an atmosphere containing 5% CO_2_. 6-8 week-old female Balb/c mice were purchased from The Jackson Laboratory (Bar Harbor, Maine). AB12 cells (2×10^6^) were injected subcutaneously (sc) into the right hind leg as primary local tumor (referred to as Tumor 1, T1). The right flank was used for the injection of a second tumor (T2) to test the immunogenic effect induced by LRT of T1. T2 was injected sequentially or concurrently with T1. When tumors grew to the designated size (5mm in diameter), animals were randomly divided into groups for treatment. Mice with overly large or small tumors were excluded. Maximal diameter of T1 and perpendicular diameters (volume) of T2 were measured twice weekly to evaluate the effect on tumor growth.

### *In vitro* cytotoxicity of γ-rays on AB12 cells determined by clonogenic assay and flow cytometry

AB12 cells (2×10^6^ /10ml tube) were exposed to different doses of γ-rays by Cs-137 Gamma Cell Irradiator-40 (Atomic Energy of Canada Ltd, Ottawa, Canada) at a dose rate of approximately 100cGy/min. After treatment with doses of 0, 1, 2, 5, 10, 15 and 20Gy, the cells were diluted serially and seeded into triple wells of 12-well plates for 10 day culture in complete medium. Surviving fraction was determined by clonogenic assay [36].

Cells were stained with Annexin V conjugated with FITC for early apoptosis and fixable viability dye Fluor-450 or propidium iodide (eBioscience) for dead cells, following the instructions provided by the manufacturer. Cellular apoptosis and death induced by radiation were assessed by flow cytometry.

### Immune responses induced by irradiated AB12 cells as an in situ vaccine to mimic local radiotherapy in mice

Irradiated AB12 cells (2×10^6^) with 0, 5, and 15Gy were injected sc into the right flank, whereas the same number of untreated cells were injected into the left flank of mice. Tumor growth curves were plotted according to the maximal perpendicular diameters as a function of days after tumor challenge.

In a separate experiment, when mice were sacrificed on day 10 after tumor cell injection, tumors, draining lymph nodes and spleen were collected for assessment of T cell subsets and activation by flow Cytometry.

### Treatment schedule of local radiotherapy followed by systemic CTLA-4 blockade

When tumors grew to a certain size in volume, local radiotherapy (LRT) was delivered with Cs-137 Gamma Cell Irradiator-40. Mice were anaesthetized with an intraperitoneal (ip) injection of 1.25% avertin (240mg/kg, Sigma) 300μl per mouse. The tumor-bearing leg was exposed to LRT, and the rest of the body was protected with a lead shielding chamber (MarsMetal Co, Burlington, ON) with 1 inch depth for each wall. Tumor-bearing mice were divided into groups as follows:

### The effect of LRT on the growth of primary T1 and secondary T2 tumors-Sequential model

1) T1-T2: The primary tumor (T1) was inoculated into the right hind leg on day 0, and the secondary tumor (T2) was injected into the right flank 10 days later. This group had no treatment and was used as control.

2) T1-LRT-T2: T1 and T2 were injected on day 0 and 10 respectively. Local radiotherapy (LRT) 5Gy was delivered to T1 once daily for 3 consecutive days, ie, day 5, 6 and 7 after tumor challenge.

3) T1-LRT-Anti-CTLA4-T2: T1 and T2 were injected on day 0 and 10 respectively. LRT 5Gy was delivered once daily for 3 consecutive days on days 5, 6 and 7. Anti-mouse CTLA-4 monoclonal antibody 100μg/mouse was injected ip twice (days 8 and 9).

### The effect of LRT on the growth of local and distant tumors-Concurrent model

4) T1T2: The local tumor (T1) and secondary tumor (T2) were injected on the same day (day 0) into the right thigh and flank, respectively. No treatment was delivered as a control.

5) T1T2-LRT: T1 and T2 were injected concurrently (day 0), and LRT 5Gy was delivered to T1 once daily for 3 consecutive days on days 5, 6 and 7 after tumor challenge.

6) T1T2-LRT-Anti-CTLA4: T1 and T2 were injected concurrently (day 0), LRT 5Gy was delivered once daily for 3 days (day 5, 6 and 7). Anti-mouse CTLA-4 monoclonal antibody 100μg/mouse was injected ip twice (day 8 and 9). Treatment schema was indicated in Fig. 1.

To test the role of the immune system, the non-obese diabetes/severe combined immune deficient (NOD/SCID) mice were used as controls. Mice were provided by Max Bell Animal Centre of University Health Network, Toronto, ON. The same number of AB12 cells (2×10^6^) was injected sc into the right thigh (T1) and flank (T2) on the same day. The tumor-bearing leg (T1) of NOD/SCID mice was also exposed to LRT 5Gy, to observe any potential effect on the distant tumor growth in the flank (T2).

A separate experiment was used as another control. Tumor cells were injected into the right flank only (T2), while no tumor was challenged on the leg (T1 free). One group of T1-free leg was treated with LRT only while the other group of T1-free leg was left untreated to compare the bystander effect on the distant tumor (T2).

### Chracterization of tumor-infiltrating T cells by fluorescent microscopy

At 10days after completion of treatment, tumors were dissected from mice of each group and embedded into OCT (Sakura, Japan) and snap frozen in liquid nitrogen. The tumor blocks were kept in −80°C freezer until sectioning was performed. Sections (5μm) were cut and stained with anti-mouse CD3-Alexa555 (eBioscience), anti-mouse-CD31-Alexa488 (Abcam) and DAPI (Cell Signaling) following the commercial instructions. The fluorescence images of whole slides were captured by an Olympus Upright microscope (Tokyo, Japan) with an automated stage and Metamporh software (Sunnyvale, CA).

### Tumor-infiltrating Tregs and T cells determined by flow cytometry

On day 7 after completing treatment, spleens and draining lymph nodes were removed from tumor-bearing mice and placed into ice-cold RPMI1640 medium containing 1% FBS. Peripheral blood was drawn from the heart of mice that were immediately euthanized by inhalation of CO_2_. Homogenized spleen and lymph node were passed through the Cell Strainer to achieve single cells. ACK lysis buffer (Invitrogen, Carlsbad, CA) was added and allowed to react for at least 15min at RT to lyse red blood cells. After washing thrice with staining buffer, appropriate dilutions (1:50~100) of Abs or isotype controls were added to each tube, 15min at RT in the dark. Staining of surface markers including CD3, CD4, CD8, CD69, CD25 and ICOS were washed thrice with staining buffer and resuspended in 1% paraformadehyde/PBS (v/v) (Sigma). After fixation with 1% paraformadehyde at 4^o^C overnight, cells were permeabilized for intracellular staining of IFN-γ, FoxP3, perforin and granzyme B and fixed with 200μl Permeabilization/Fixation Solution (eBioscience), and then washed twice with permeabilization buffer. Anti-mouse antibodies against IFN-γ (1:30), perforin (1:50) and granzyme B (1:50) were added and maintained at RT for 20min in the dark.

Single cell suspensions were stained with monoclonal antibodies conjugated with different fluorescent dyes, CD3 (clone: 17A2)-PE-Cy7, CD4 (clone: RM4-5)-FITC, CD8β (clone: H35-17.2)-APC, CD69 (clone: H1.2F3)-FITC, CD25 (clone: PC61.5)-APC, ICOS (clone: 15F9)-PE, IFN-γ (clone: XMG1.2)-PE, perforin (clone: eBioOMAK-D)-FITC, FoxP3 (clone: FJK-16s)-PE, and granzyme B (clone:16G6)-PE. All antibodies and isotypes were purchased from eBioscience or BioLegend (San Diego, CA). Becton Dickinson LSR II Flow Cytometer (San Jose, CA) and FACSDiva^TM^ software were used for data acquisition ^TM^ and FlowJo^TM^ software was used for analysis.

### Expression of the immune-associated genes analyzed by RT-PCR

Total RNA was extracted from tumors and spleens using TRizol Reagent (Invitrogen), and RNeasy MinElute Cleanup kit (QIAGEN, Valencia, CA) enabled cleanup of RNA. cDNA was synthesized with High-Capacity cDNA Reverse Transcription kits (ABI, Foster City, CA) on a PTC-100^TM^ Programmable Thermal controller (MJ research Inc., Gaithersburg, MD) following the manufacturer's protocols. Regular PCR was carried out to establish RT-PCR standards of all target genes including CD3, CD4, CD8, ICOS, IFN-γ, granzyme B, perforin, TNF-α, IP-10, CD80, CD86 and house-keeping gene GAPDH. DNA fragments were obtained from regular PCR on a PTC-100^TM^ Programmable Thermal. Regular PCR was performed by 10× High Fidelity PCR Buffer, Platinum® Taq polymerase High Fidelity, 50mM MgSO_4_, 10mM dNTP Mix (Invitrogen). A SYBR GREEN real-time PCR was performed on ABI PRISM 7900HT system. PCR was composed of Power SYBR^TM^ GREEN PCR 2× Master Mix (ABI), 200nM primer and 2μl 500ng/μl cDNA ×40 cycles. Primers of house-keeping and all target genes were designed by using ABI Prism® Primer Express^TM^software Version 2.0.

### Cytokine profile determined by Luminex assay

Culture medium of splenocytes derived from different groups was collected for cytokine detection by the Luminex assay. Assay was performed in magnetic plate and according to BioRad Mouse Cytokine/Chemokine protocol (The Princess Margaret Genomics Centre, Toronto, ON). 50μl beads were washed twice with 100μl of wash buffer and 50μl sample was added and incubated for 30min at RT. Beads were subsequently washed thrice and incubated with 25μl of detection antibody for 30min. 50μl streptavidin-phycoerthyrin was added to the beads for 10min at RT. Beads were again washed and resuspended in 125μl of assay buffer and read with Luminex-100 and data were analyzed using Bio-plex Manager 6.0. All the concentrations of cytokines are presented in unit of pg/ml.

### Statistical analysis

All data are presented as the mean ± SEM. The comparison of cytokine gene expression and proportion of T cell subsets between two groups was analyzed by using an unpaired Student's *t* test. ANOVA was performed when compared among multiple groups using GraphPad Prism 6.0 statistical software (La Jolla, CA). A value of *P* < 0.05 was considered significantly different for all comparisons. * *P* < 0.05; ** P < 0.01; *** P < 0.001.

## SUPPLEMENTARY MATERIAL, FIGURES



## References

[1] Zauderer MG, Krug LM (2011). The evolution of multimodality therapy for malignant pleural mesothelioma. Curr Treat Options Oncol.

[2] Tonoli S, Vitali P, Scotti V, Bertoni F, Spiazzi L, Ghedi B, Buonamici FB, Marrazzo L, Guidi G, Meattini I (2011). Adjuvant radiotherapy after extrapleural pneumonectomy for mesothelioma. Prospective analysis of a multi-institutional series. Radiother Oncol.

[3] Gomez DR, Hong DS, Allen PK, Welsh JS, Mehran RJ, Tsao AS, Liao Z, Bilton SD, Komaki R, Rice DC (2013). Patterns of failure, toxicity, and survival after extrapleural pneumonectomy and hemithoracic intensity-modulated radiation therapy for malignant pleural mesothelioma. J Thorac Oncol.

[4] Buduhan G, Menon S, Aye R, Louie B, Mehta V, Vallieres E (2009). Trimodality therapy for malignant pleural mesothelioma. Ann Thorac Surg.

[5] de Perrot M, Feld R, Cho BC, Bezjak A, Anraku M, Burkes R, Roberts H, Tsao MS, Leighl N, Keshavjee S (2009). Trimodality therapy with induction chemotherapy followed by extrapleural pneumonectomy and adjuvant high-dose hemithoracic radiation for malignant pleural mesothelioma. J Clin Oncol.

[6] Hiddinga BI, van Meerbeeck JP (2013). Surgery in mesothelioma--where do we go after MARS?. J Thorac Oncol.

[7] Pasello G, Ceresoli GL, Favaretto A (2013). An overview of neoadjuvant chemotherapy in the multimodality treatment of malignant pleural mesothelioma. Cancer Treat Rev.

[8] Stamell EF, Wolchok JD, Gnjatic S, Lee NY, Brownell I (2013). The abscopal effect associated with a systemic anti-melanoma immune response. Int J Radiat Oncol Biol Phys.

[9] Demaria S, Formenti SC (2012). Role of T lymphocytes in tumor response to radiotherapy. Front Oncol.

[10] Frey B, Rubner Y, Wunderlich R, Weiss EM, Pockley AG, Fietkau R, Gaipl US (2012). Induction of abscopal anti-tumor immunity and immunogenic tumor cell death by ionizing irradiation - implications for cancer therapies. Curr Med Chem.

[11] Burnette BC, Liang H, Lee Y, Chlewicki L, Khodarev NN, Weichselbaum RR, Fu YX, Auh SL (2011). The efficacy of radiotherapy relies upon induction of type i interferon-dependent innate and adaptive immunity. Cancer Res.

[12] Suzuki Y, Mimura K, Yoshimoto Y, Watanabe M, Ohkubo Y, Izawa S, Murata K, Fujii H, Nakano T, Kono K (2012). Immunogenic tumor cell death induced by chemoradiotherapy in patients with esophageal squamous cell carcinoma. Cancer Res.

[13] Wersall PJ, Blomgren H, Pisa P, Lax I, Kalkner KM, Svedman C (2006). Regression of non-irradiated metastases after extracranial stereotactic radiotherapy in metastatic renal cell carcinoma. Acta Oncol.

[14] Kingsley DP (1975). An interesting case of possible abscopal effect in malignant melanoma. Br J Radiol.

[15] Postow MA, Callahan MK, Barker CA, Yamada Y, Yuan J, Kitano S, Mu Z, Rasalan T, Adamow M, Ritter E (2012). Immunologic correlates of the abscopal effect in a patient with melanoma. N Engl J Med.

[16] Frey B, Stache C, Rubner Y, Werthmoller N, Schulz K, Sieber R, Semrau S, Rodel F, Fietkau R, Gaipl US (2012). Combined treatment of human colorectal tumor cell lines with chemotherapeutic agents and ionizing irradiation can *in vitro* induce tumor cell death forms with immunogenic potential. J Immunotoxicol.

[17] Pilones KA, Kawashima N, Yang AM, Babb JS, Formenti SC, Demaria S (2009). Invariant natural killer T cells regulate breast cancer response to radiation and CTLA-4 blockade. Clin Cancer Res.

[18] Liao YP, Wang CC, Schaue D, Iwamoto KS, McBride WH (2009). Local irradiation of murine melanoma affects the development of tumour-specific immunity. Immunology.

[19] Wu L, Yun Z, Tagawa T, Rey-McIntyre K, de Perrot M (2012). CTLA-4 blockade expands infiltrating T cells and inhibits cancer cell repopulation during the intervals of chemotherapy in murine mesothelioma. Mol Cancer Ther.

[20] Lesterhuis WJ, Salmons J, Nowak AK, Rozali EN, Khong A, Dick IM, Harken JA, Robinson BW, Lake RA (2013). Synergistic effect of CTLA-4 blockade and cancer chemotherapy in the induction of anti-tumor immunity. PLoS One.

[21] Ruocco MG, Pilones KA, Kawashima N, Cammer M, Huang J, Babb JS, Liu M, Formenti SC, Dustin ML, Demaria S (2012). Suppressing T cell motility induced by anti-CTLA-4 monotherapy improves antitumor effects. J Clin Invest.

[22] Wirsdorfer F, Cappuccini F, Niazman M, de Leve S, Westendorf AM, Ludemann L, Stuschke M, Jendrossek V (2014). Thorax irradiation triggers a local and systemic accumulation of immunosuppressive CD4+ FoxP3+ regulatory T cells. Radiat Oncol.

[23] Yoshimoto Y, Suzuki Y, Mimura K, Ando K, Oike T, Sato H, Okonogi N, Maruyama T, Izawa S, Noda SE (2014). Radiotherapy-induced anti-tumor immunity contributes to the therapeutic efficacy of irradiation and can be augmented by CTLA-4 blockade in a mouse model. PLoS One.

[24] Ozsahin M, Crompton NE, Gourgou S, Kramar A, Li L, Shi Y, Sozzi WJ, Zouhair A, Mirimanoff RO, Azria D (2005). CD4 and CD8 T-lymphocyte apoptosis can predict radiation-induced late toxicity: a prospective study in 399 patients. Clin Cancer Res.

[25] Manda K, Glasow A, Paape D, Hildebrandt G (2012). Effects of ionizing radiation on the immune system with special emphasis on the interaction of dendritic and T cells. Front Oncol.

[26] Rubner Y, Wunderlich R, Ruhle PF, Kulzer L, Werthmoller N, Frey B, Weiss EM, Keilholz L, Fietkau R, Gaipl US (2012). How does ionizing irradiation contribute to the induction of anti-tumor immunity?. Front Oncol.

[27] Rodel F, Frey B, Multhoff G, Gaipl U (2015). Contribution of the immune system to bystander and non-targeted effects of ionizing radiation. Cancer Lett.

[28] Cho BC, Feld R, Leighl N, Opitz I, Anraku M, Tsao MS, Hwang DM, Hope A, de Perrot M (2014). A Feasibility Study Evaluating Surgery for Mesothelioma After Radiation Therapy: The “SMART” Approach for Resectable Malignant Pleural Mesothelioma. J Thorac Oncol.

[29] Al-Taei S, Salimu J, Lester JF, Linnane S, Goonewardena M, Harrop R, Mason MD, Tabi Z (2012). Overexpression and potential targeting of the oncofoetal antigen 5T4 in malignant pleural mesothelioma. Lung Cancer.

[30] Formenti SC, Demaria S (2013). Combining radiotherapy and cancer immunotherapy: a paradigm shift. J Natl Cancer Inst.

[31] Kaminski JM, Shinohara E, Summers JB, Niermann KJ, Morimoto A, Brousal J (2005). The controversial abscopal effect. Cancer Treat Rev.

[32] Khan MA, Van Dyk J, Yeung IW, Hill RP (2003). Partial volume rat lung irradiation; assessment of early DNA damage in different lung regions and effect of radical scavengers. Radiother Oncol.

[33] Calveley VL, Khan MA, Yeung IW, Vandyk J, Hill RP (2005). Partial volume rat lung irradiation: temporal fluctuations of in-field and out-of-field DNA damage and inflammatory cytokines following irradiation. Int J Radiat Biol.

[34] Lipson EJ (2013). Re-orienting the immune system: Durable tumor regression and successful re-induction therapy using anti-PD1 antibodies. Oncoimmunology.

[35] Ott PA, Hodi FS, Robert C (2013). CTLA-4 and PD-1/PD-L1 blockade: new immunotherapeutic modalities with durable clinical benefit in melanoma patients. Clin Cancer Res.

[36] Fung AS, Wu L, Tannock IF (2009). Concurrent and sequential administration of chemotherapy and the Mammalian target of rapamycin inhibitor temsirolimus in human cancer cells and xenografts. Clin Cancer Res.

