# Supplementary material for: Targeting the inhibitory receptor CTLA-4 on T cells increased abscopal effects in murine mesothelioma model
Source: Oncotarget. 2015 Mar 8;6(14):12468–80. doi: 10.18632/oncotarget.3487 (PMC4494951; doi:10.18632/oncotarget.3487)
Supplement: Supplementary file 1 [file oncotarget-06-12468-s001.pdf]

Targeting the inhibitory receptor CTLA-4 on T cells increased abscopal effects in murine mesothelioma model

Supplementary Material

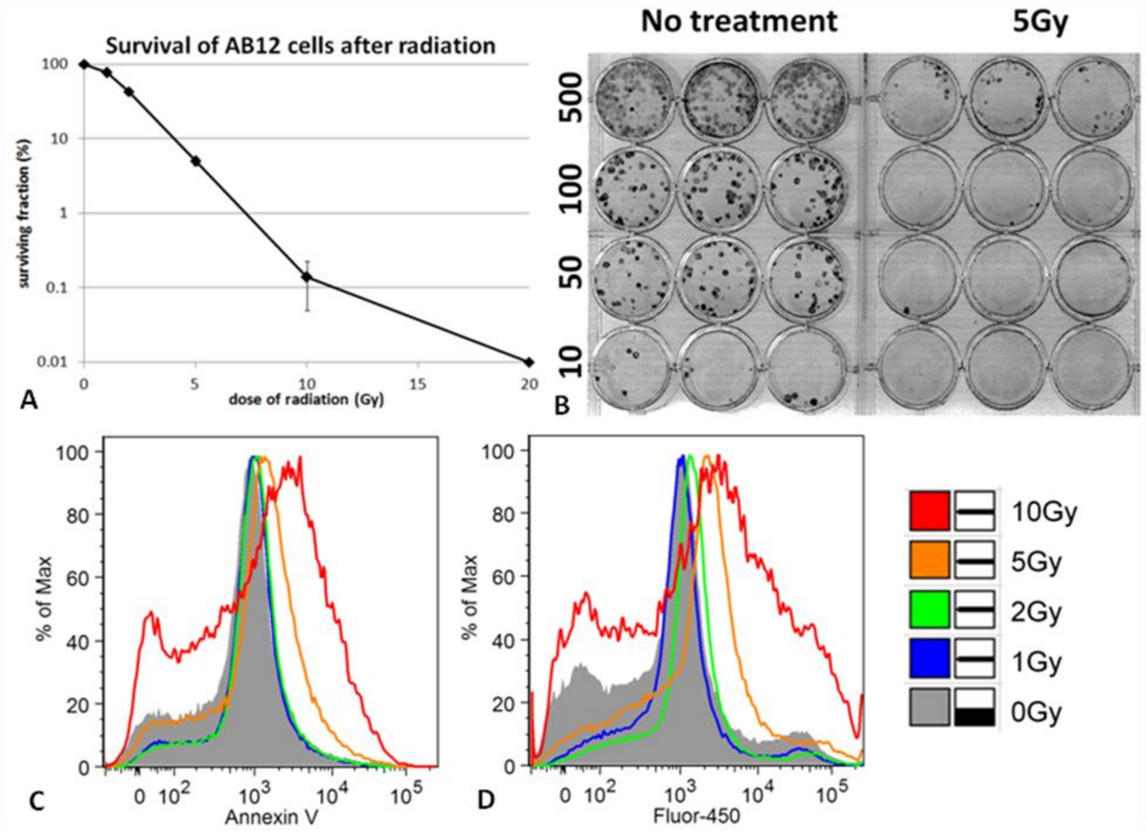

Supplementary figure 1

In vitro cytotoxicity on AB12 cells after exposure to different doses of γ–ray radiation. A) Surviving fraction of AB12 cells after exposure to different doses of γ-rays ranging from 1Gy to 20Gy was determined by clonogenic assay; B) Colonies were shown at the end of 10-day culture after exposure to 5Gy radiation; C) Cellular apoptosis and D) death were determined by Annexin V and Flour-450 staining, respectively, after radiation.

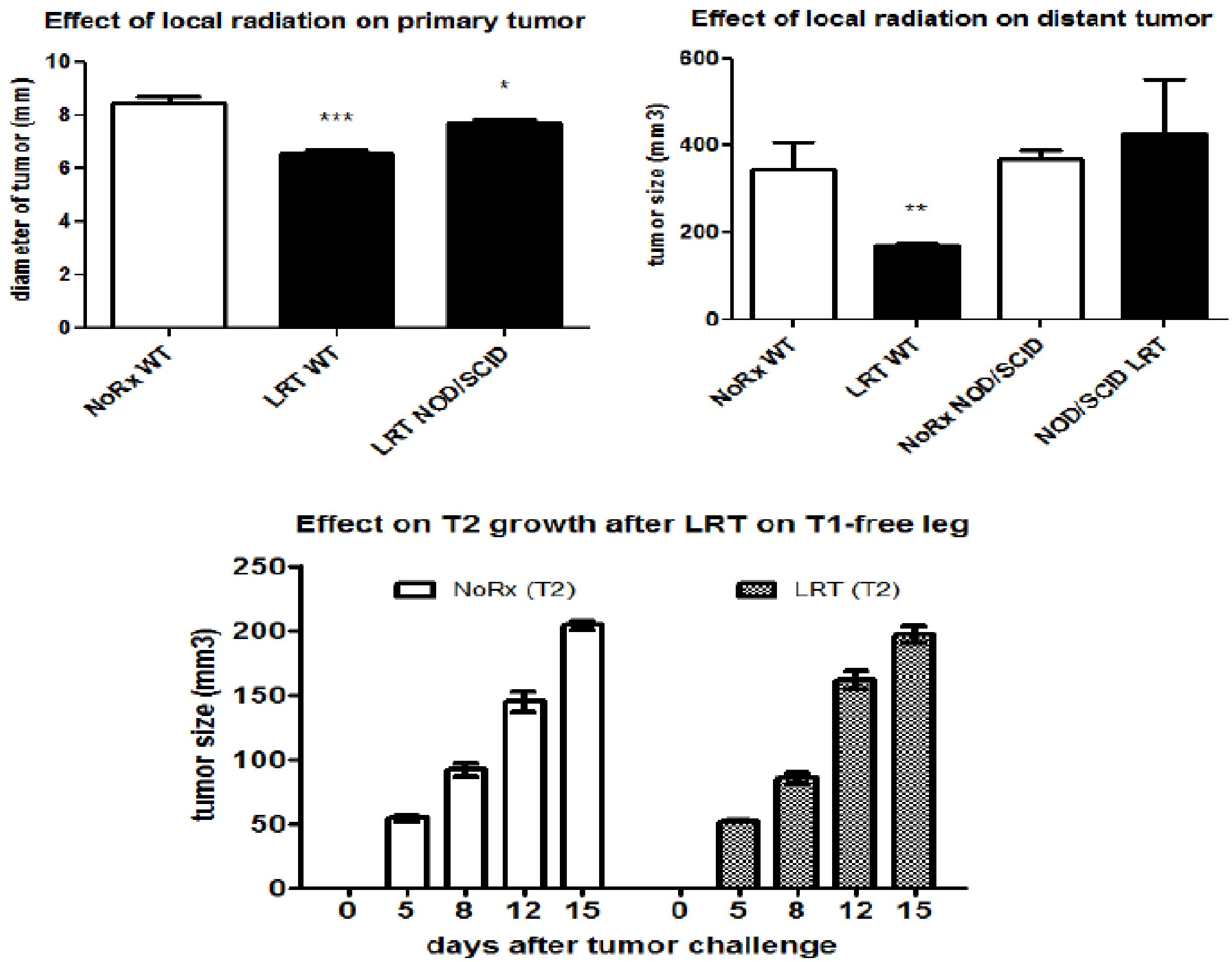

Supplementary figure 2

Effect of local tumor radiation on the distant tumor growth (T2) in the NOD/SCID mice (top panel); and the effect of local radiation on tumor-free leg on the distant tumor growth (T2) in Balb/c wild-type mice (bottom).

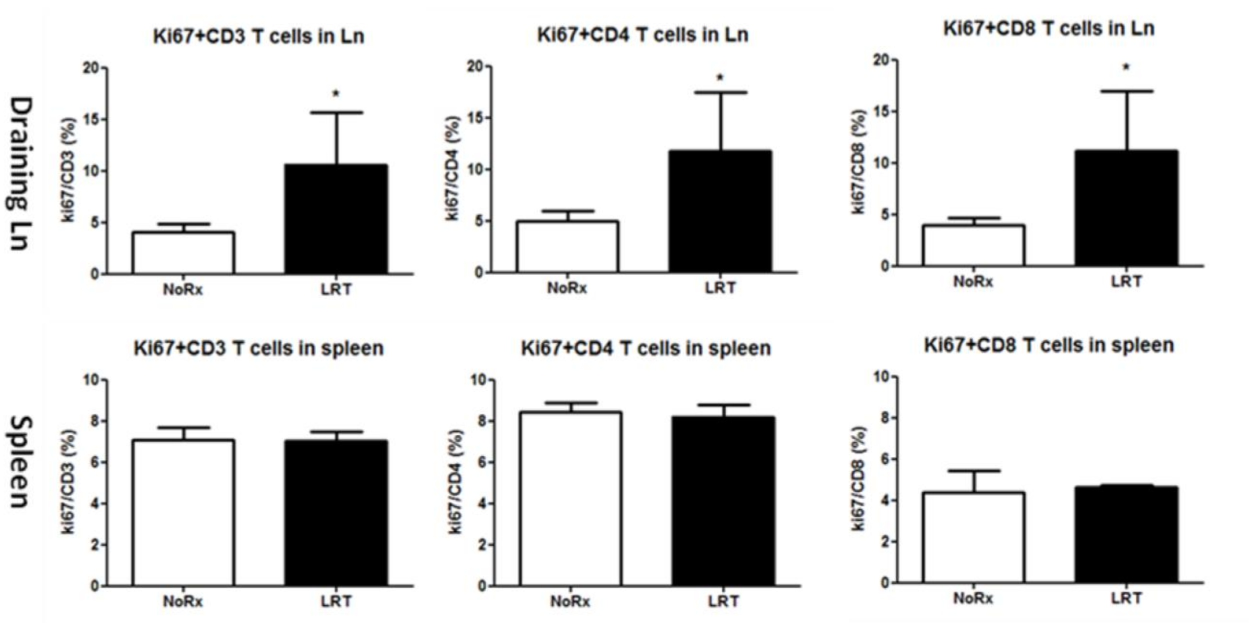

Supplementary figure 3

Proliferation of T cells in the draining lymph nodes (top panel) was dramatically increased in the radiated group, while no significant difference was observed in the spleen (bottom panel).

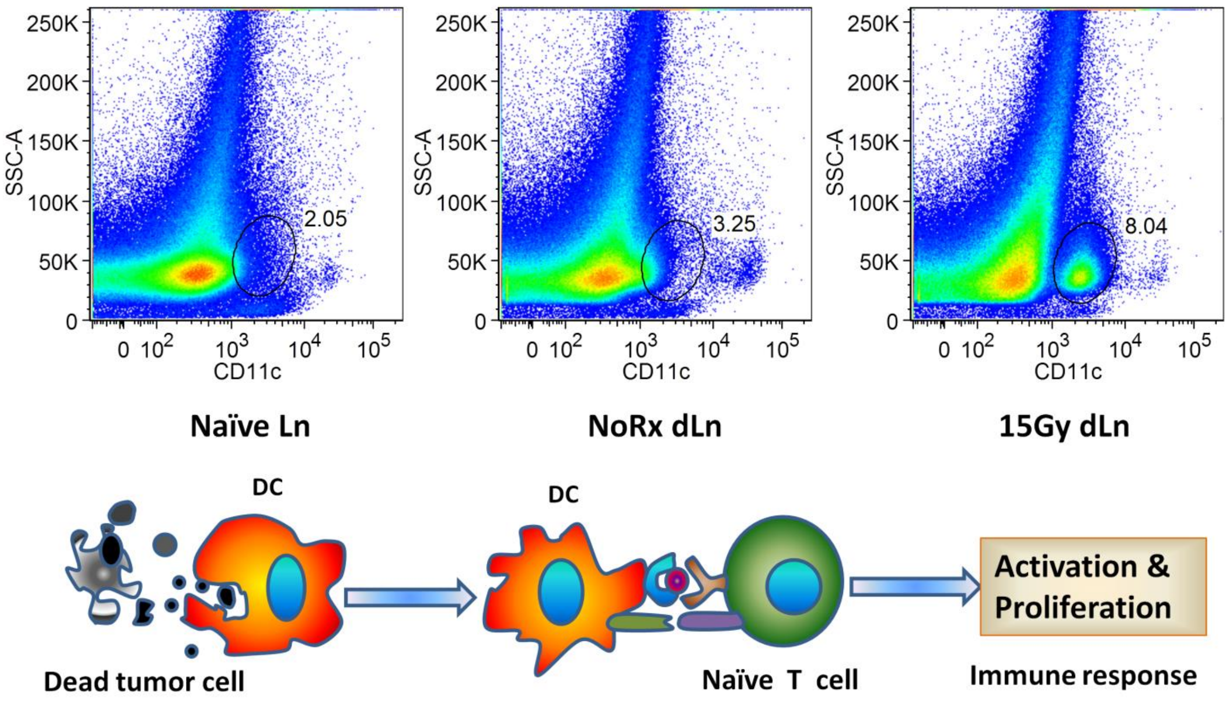

Supplementary figure 4

On day 3 after injection of the irradiated AB12 cells, the proportion of dendritic cells in the draining lymph nodes was found to increase.
